# Do stress hormones influence choice? A systematic review of pharmacological interventions on the HPA axis and/or SAM system

**DOI:** 10.1093/scan/nsae069

**Published:** 2024-10-04

**Authors:** Luis Felipe Sarmiento, Jorge Alexander Ríos-Flórez, Fabio Alexis Rincón Uribe, Rafael Rodrigues Lima, Tobias Kalenscher, Amauri Gouveia, Felix Jan Nitsch

**Affiliations:** BiotechMed Center, BME Lab, Multimedia Systems Department, Faculty of Electronics, Telecommunications, and Informatics, Gdansk University of Technology, Gdansk 80-233, Poland; Professor at the Faculty of Law and Forensic Sciences, Tecnológico de Antioquia University Institution, Medellín 050034, Colombia; Department of postgraduation in Psychology, Federal University from Pará, Belém 66075-110, Brazil; Laboratory of Functional and Structural and Biology, Federal University from Pará, Belém 66075-110, Brazil; Comparative Psychology, Institute of Experimental Psychology, Heinrich Heine University Düsseldorf, 40225, Germany; Laboratory of Neuroscience and Behavior, Federal University from Pará, Belém 66075-110, Brazil; Marketing Area, INSEAD, Fontainebleau 77300, France; Paris Brain Institute (ICM), Sorbonne University, Paris 75013, France

**Keywords:** cortisol, decision-making, noradrenaline, stress, HPA axis, SAM system

## Abstract

The hypothalamus–pituitary–adrenal axis (HPA axis) and the sympathetic–adrenal–medullary system (SAM system), two neuroendocrine systems associated with the stress response, have often been implicated to modulate decision-making in various domains. This systematic review summarizes the scientific evidence on the effects of pharmacological HPA axis and SAM system modulation on decision-making. We found 6375 references, of which 17 studies fulfilled our inclusion criteria. We quantified the risk of bias in our results with respect to missing outcome data, measurements, and selection of the reported results. The included studies administered hydrocortisone, fludrocortisone (HPA axis stimulants), yohimbine, reboxetine (SAM system stimulants), and/or propranolol (SAM system inhibitor). Integrating the evidence, we found that SAM system stimulation had no impact on risk aversion, loss aversion or intertemporal choice, while SAM system inhibition showed a tentative reduction in sensitivity to losses. HPA axis stimulation had no effect on loss aversion or reward anticipation but likely a time-dependent effect on decision under risk. Lastly, combined stimulation of both systems exhibited inconsistent results that could be explained by dose differences (loss aversion) and sex differences (risk aversion). Future research should address time-, dose-, and sex-dependencies of pharmacological effects on decision-making.

## Introduction

Do you want to quit your job? Do you want to propose to your girlfriend? And what about the choice of a name for your child? Some decisions are difficult and not easily accessible to a simple cost-benefit analysis. A prominent lay theory says that bodily sensations guide such choices (i.e. the gut feeling): If the thought of a decision makes you uncomfortable or anxious this might indicate that you do not like to choose this way.

Decision neuroscience formalized this idea in the Somatic Marker Hypothesis, which posits that decision-makers infer choice value from somatic reactions, such as changes in their heart rate or endocrine levels (Damasio 1994). But, despite its intuitive appeal, the causal impact of physiological systems on decision-making remains an active area of research and it is often not clear how our body influences our choices. One reason for this is that many physiological processes present a complex and orchestrated interplay of multiple systems. Prominently, the acute stress response plays out over multiple distinct physiological systems and has been suggested to have opposite effects on behavior depending on timing, individual differences, and the relative activation of each system.

The current systematic review, therefore, revisits experiments using causal and specific pharmacological interventions to study the role of two major neuroendocrine systems in decision-making: the hypothalamic–pituitary–adrenal (HPA) axis and the sympathetic–adreno–medullary (SAM) system. This approach is admittedly reductionist and limited in ecological validity, but a priori appears most likely to produce internally consistent results. Concretely, we summarize the current state of evidence, make sense of overt inconsistencies, and identify concrete pathways for future research. The HPA axis is a major neuroendocrine system intricately connected to various physiological processes, including digestion, immune system function, mood, and emotion, as well as energy storage and expenditure. Similarly, the SAM system plays a crucial role in regulating the physiological response to external stimuli, exemplified by the “fight or flight” response, and has been linked to cognition, emotion, and attention. Both systems contribute to the dynamic regulation of behavior in response to environmental challenges, in particular during the stress response, reactions to external threats, or in the context of reward-related behavioral pathologies. Therefore, they have received pronounced interest in decision neuroscience.

In the following sections, we provide an overview of both neuroendocrine systems, with a focus on their effect on the central nervous system and executive functions related to decision-making. We further provide an overview of drugs that act on these systems to contextualize the reviewed pharmacological protocols.

### The HPA axis

The HPA axis is a complex neuroendocrine system crucial for maintaining physiological homeostasis. Originating in the hypothalamus, corticotropin-releasing hormone initiates a cascade reaction, leading to the release of adrenocorticotropic hormone from the pituitary gland. This, in turn, stimulates the adrenal glands to produce corticosteroids, including cortisol. The main function of the HPA axis is to modulate various physiological processes such as the body’s response to stress, immunity, metabolism, fertility, and the autonomic nervous system (Demorrow 2018).

In the brain, cortisol primarily binds to mineralocorticoid receptors (MRs) and glucocorticoid receptors (GRs), which cooperate in the mediation of cortisol effects. MRs and GRs have a 10-fold difference in their affinity for binding cortisol. MRs have a relatively high affinity, such that even low levels of cortisol (e.g. under rest) lead to substantial occupation of the receptors in the coordination of circadian events, stress-coping, and adaptation. MRs serve as the on-switch; they are relevant for the tone, threshold, and sensitivity of the response system. In contrast, GRs have a much lower affinity and, therefore, are mostly only occupied by cortisol during circadian hormonal peaks and during the stress response. GRs, conversely, serve as the off-switch of the response system; GR-mediated actions modulate the duration and termination of cortisol secretion. Both, MR- and GR-mediated actions involve rapid non-genomic and slower gene-mediated mechanisms (de Kloet and Joëls 2024).

The HPA axis also interacts with neurotransmitter systems in the brain, particularly those involving serotonin and dopamine. Serotonin, often associated with mood regulation, and dopamine, implicated in reward and pleasure pathways, play crucial roles in shaping emotional and cognitive states (Smith and Vale 2006).

Various drugs exist that modulate the HPA axis. For example, drugs like hydrocortisone and fludrocortisone stimulate the HPA axis. Hydrocortisone increases cortisol levels, exhibiting anti-inflammatory and immunosuppressive effects. Fludrocortisone, a synthetic mineralocorticoid, is used to replace aldosterone in adrenal insufficiency (Russell and Lightman 2019). Conversely, spironolactone inhibits the HPA axis by acting as an aldosterone receptor antagonist (Gómez-Sánchez 2016).

#### HPA axis and cognitive function

Multiple lines of work implicate the crucial role of the HPA axis in healthy and impaired cognitive function. For example, acute cortisol effects have been linked to impaired memory retrieval (Buchanan and Tranel 2008). The ability to retrieve and integrate past experiences may in turn influence how individuals assess and make decisions. Query Theory (Weber et al. 2007) asserts that preferences are not always fully innate but instead may be constructed during choice, by retrieving relevant experiences from memory. Cortisol’s influence also extends to attentional processes, playing a pivotal role in shaping cognitive focus and vigilance. Like memory, attention is an executive function fundamental to choose according to pervasive theoretical accounts (e.g. Smith and Ratcliff 2009, Krajbich 2019). Attention guides which choice options and attributes are intended to and might influence valuation itself.

Conditions such as Cushing’s syndrome, characterized by prolonged hypercortisolism, for example, often result in neuropsychiatric disorders. Memory, attention, and executive functions are commonly impaired, and these cognitive deficits persist, even after treatment, potentially due to glucocorticoid-dependent structural alterations in the brain (Pivonello et al. 2015). Such cortisol-driven changes to memory systems lead to a dominance of more automatic and habit-based responses over flexible cognitive processing (Zerbes et al. 2022). Further, excessive cortisol levels have been linked to impairments in sustained attention, selective attention, and working memory, particularly affecting the prefrontal cortex, a brain region crucial for attention and executive functions (Liu et al. 2020).

Adrenal insufficiency, marked by cortisol deficiency, is linked to cognitive deficits, particularly affecting declarative memory and processing speed (Matsuo et al. 2020). Similarly, congenital adrenal hyperplasia, a genetic disorder impacting adrenal steroidogenesis, is associated with lower intelligence quotient and cognitive impairment, particularly in visual perception and executive functioning (Johannsen et al. 2006). Additionally, the administration of exogenous glucocorticoids, a common treatment for autoimmune and inflammatory disorders, has been linked to cognitive deficits (Judd et al. 2014). These cognitive effects are marked by hippocampal, amygdala, and prefrontal cortex structural alterations, suggesting the complex and multifaceted nature of the relationship between glucocorticoids and cognitive function (McEwen et al. 2016). The intricate interplay between cortisol and cognitive function underscores the dual nature of the HPA axis physiology, where optimal levels may enhance attentional focus, but chronic cortisol dysregulation may contribute to various cognitive deficits.

The direct role of the HPA axis and cortisol in decision-making has been predominantly studied within the context of the acute stress response (see Duque et al. 2022 for a review). However, as outlined above, the role of the HPA axis extends much further than the stress response.

### The SAM system

The SAM system is a crucial component of the sympathetic nervous system, facilitating rapid physiological adaptation in response to various challenges. This system orchestrates short-lasting responses such as heightened alertness, increased vigilance, and rapid appraisal of the surrounding situation. Its physiological reactions are mediated by catecholamines, notably adrenaline and noradrenaline (NA), primarily secreted from the adrenal medulla (Tank and Wong 2011).

Adrenaline, a key player in the SAM system, activates vagal afferents that terminate on noradrenergic cells within the nucleus of the solitary tract. These noradrenergic projections influence NA release, acting through direct or indirect pathways to the locus coeruleus (LC). The LC serves as a primary source for an extensive network of NA projections, encompassing critical brain regions such as the hippocampus, amygdala, and neocortex (McGaugh and Roozendaal 2002, van Stegeren 2008, Ness and Calabrese 2016).

The adrenergic pathway within the SAM system involves three families of adrenergic receptors (AR)—α1, α2, and β—with a total of nine subtypes. These receptors are expressed in the brain, exhibiting distinct expression patterns, signal transduction pathways, and physiological regulations that profoundly affect neuronal firing and excitability. Notably, the LC regulates neuronal function through α- and β-adrenergic receptors, interacting extensively within the central nervous system (Timmermans et al. 2013).

The release of adrenaline and noradrenaline has various physiological effects, including increasing heart rate and blood pressure, enhancing alertness, learning, and memory. It prepares the body for a “fight or flight” response and maintains alertness, with metabolic actions such as increasing glucose via glycogenolysis and gluconeogenesis, and cardiovascular actions including lipolysis, increased oxygen consumption, and thermogenesis (Chu et al. 2019).

The SAM system also serves as a dynamic regulator of various physiological functions beyond its renowned role in the stress response. One notable function is its impact on cardiovascular dynamics, influencing the heart rate to ensure an efficient supply of oxygen to vital organs. The SAM system also orchestrates the dilation of pupils, optimizing visual acuity in response to environmental stimuli. Simultaneously, it promotes bronchodilation, facilitating increased airflow to the lungs to enhance oxygen intake during heightened activity (Bradley et al. 2017).

In pharmacological research to study the SAM system, drugs such as yohimbine, reboxetine, and propranolol are utilized. Yohimbine, an alpha 2-selective agonist, blocks alpha-2 receptors, increasing firing of noradrenergic neurons in the LC and the release of noradrenaline. Reboxetine, a selective noradrenaline reuptake inhibitor, increases extracellular noradrenaline availability by blocking reuptake. Propranolol, a non-selective beta-adrenergic blocker, is used for various conditions, including cirrhosis, esophageal varices, hypertension, and ischemic heart disease, by reducing blood pressure and heart rate (McGaugh and Roozendaal 2002, van Stegeren 2008, Timmermans et al. 2013, Ness and Calabrese 2016).

#### SAM system and cognition

Like for the HPA axis, there is mounting evidence that implicates the SAM system in healthy and impaired cognition. Most prominently, the noradrenaline system plays a pivotal role in driving shifts in attention and to enhance behavioral performance. Animal research shows that certain levels of noradrenaline in the prefrontal cortex are required for selective attention and working memory tasks as indicated by performance drops under pharmacological inhibition (Sara 2009). The release of adrenaline and noradrenaline is further associated with increased arousal and response readiness. This heightened state of arousal, for example, serves as an adaptive response to stressful situations. Noradrenaline also seems to play a role for memory. In rodents, adrenergic signaling is critical for the retrieval of short-term contextual information.

Dysregulated dopaminergic and noradrenergic neurotransmission is an important characteristic in the pathophysiology of attention-deficit/hyperactivity disorder, which is characterized by symptoms of inattention, impulsivity, and/or hyperactivity. It is further associated with impairments in high-level cognitive functions such as working memory and inhibitory response control. Further, the overwhelming majority of pharmacological treatment options affect noradrenaline (see Del Campo et al. 2011).

As for the HPA axis, the SAM system has received substantial interest in decision-making research in the context of the stress response, for example via peripheral-physiological measures of arousal. In contrast, the current review focuses on direct, pharmacological manipulations of the SAM system to better understand its distinct role in the decision-making.

### The stress response

Acute stress is commonly defined as the reaction of an organism to a threat or demand exceeding its perceived capabilities to cope (Lazarus and Folkman 1984). The stress reaction serves to prepare the organism to overcome such challenges via the reallocation of resources toward immediate action (often coined the fight-or-flight response). The physiological stress response presents a complex and orchestrated interplay of multiple hormonal systems (Joëls and Baram 2009). Initially, exposure to a stressor triggers a rapid surge in central catecholamine levels, providing an immediate physiological response. In contrast, corticosteroid levels in the brain rise more gradually and sustain elevation over an extended period. Catecholamines exert immediate effects on stress response, while corticosteroids exhibit a combination of rapid non-genomic and slower genomic effects. These processes may overlap and interact during an early time window following stress onset. Subsequently, different waves of stress-related neurotransmitters and hormones exert varying impacts on widely distributed brain regions. At the cellular level, catecholamines may interact with the early non-genomic effects of corticosteroids, while the genomic effects of cortisol typically manifest approximately 60 min after the onset of stress (Hermans et al. 2014).

The cognitive effects of the acute stress support the quick and efficient response to immediate demands or threats. The activation of the SAM axis during acute stress facilitates alertness and enables individuals to focus sharply on the present challenges, optimizing their cognitive resources for swift and precise responses (Berridge 2008). Adrenaline and noradrenaline play a key role in shaping the consolidation of memories, especially those linked to a stressor (Liang et al. 1986, Kilpatrick and Cahill 2003). These adaptations are further supported by the activation of the HPA axis; moderate cortisol levels during acute stress have been likewise associated with enhanced attention and increased vigilance. Further, results from animal studies suggest that periodic elevations in glucocorticoids may enhance focused attention toward an emotionally arousing stimulus (Erickson et al. 2003).

Importantly, the acute stress response involves the activation of the two neuroendocrine systems that we discuss here, the HPA axis and the SAM system. Hence, pharmacological studies intervening on either or both systems bear relevance for our understanding of stress effects as well. Specifically, the pharmacological approach has the advantage of allowing us to simulate specific facets of the physiological stress response, which potentially allows us to study the causal role of specific stress neuromodulators in cognitive functioning or decision-making. For example, hydrocortisone can be administered to mimic the hormone cortisol, one of the most important neuromodulators in the stress response; and at different doses, can stimulate the mineralocorticoid receptor and the glucocorticoid receptor differentially. Similarly, fludrocortisone binds specially to mineralocorticoid receptors. Furthermore, the administration of catecholamines and different agonists and antagonists can be used to stimulate or blockade the adrenergic receptors, emulating the effect of the catecholamines, and especially the norepinephrine system. We note that chronic stress involves other physiological processes than the acute stress response, such as the accumulation of allostatic load, which are not captured by a time-limited activation of the SAM system and/or the HPA axis (Juster et al. 2010). Hence, the pharmacological manipulations that we consider here are typically used as models of the acute stress response only. A detailed discussion of chronic stress is beyond the scope of this paper.

Social stress induction protocols such as the Trier Social Stress Test (Kirschbaum et al. 1993, Birkett 2011) and physical stress induction protocols such as the Cold Pressor Test (Lovallo 1975) on the other hand are quite unspecific from a psychophysiological perspective. Perhaps, this could provide an explanation for why proposed effects of acute stress on behavior, and especially decision-making, are heterogenous. For example, some studies found that acute stress can reduce risk-taking (Nowacki et al. 2019, Wang et al. 2022), increase risk-taking (Lighthall et al. 2009, Reynolds et al. 2013), and not influence risk-taking (Cano-López et al. 2016). Importantly, these studies all used the same acute stress protocol [the Trier Social Stress Test (TSST)], and the same risk-taking measure (the Balloon Analog Risk Taking task). However, other important moderators of the hormonal and behavioral response (e.g. timing, sex differences, and cortisol responsiveness) seem to require larger sample sizes and/or even further experimental control. Both are challenging in practice using complex stressors such as the TSST. Hence, pharmacological manipulations are often deployed and interpreted as a supplemental approach.

However, it is crucial to note that psychopharmacological manipulations of these neuroendocrine systems do not represent a fully accurate and comprehensive model of the acute stress response. The physiological stress response unfolds in distinct time windows and is more intricate than a simple HPA and SAM activation. It involves several other neuromodulators systems (e.g. serotonin, oxytocin, and opioid responses) and is influenced by context, environment, experience, and personality-dependent emotions and cognitions. Additionally, it is important to highlight that pharmacological manipulation of the HPA axis and SAM system is often not subjectively perceived as stress (see Box 1).

Therefore, pharmacological manipulations generally cannot be equated to stress, and results and conclusions should be carefully interpreted. Still, given their controlled and specific nature, we hope that a review of this literature could also shed light on the functioning of these systems in stress research and decision-making.

### Decision-making

We define decisions as the selection of one or multiple out of a set of available alternatives. Typically, decisions are thought to be based on the characteristics of the choice alternatives as well as the subjective preferences of the decision-maker. In our review, we considered various and different choice types that can be broadly categorized according to the certainty of “information available” (decisions under certainty, under risk, or under ambiguity), as well as “temporal” (immediate and/or future consequences) and “social” (social or non-social decisions) aspects. Note that the type of decisions described in the manuscript are not exhaustive, but rather reflect the current focus of the psychopharmacological literature. Specifically, so far there is psychopharmacological work on the following distinct domains.

“Decision-making under risk” is a type of choice where the outcome is uncertain. A typical metaphor (and experimental paradigm) for this type of decisions are gambles. For example, participants could be asked whether they are willing to bet on the outcome of a coin flip with a prospect of winning a larger amount of money at chance level, or they would rather receive a smaller but sure reward. Participants can then be distinguished by their trade-off between the expected value and the risk of a decision.

A concept related to the above-described decisions under risk is *loss aversion*. It can be defined as the tendency to overweigh losses, compared with gains of the same amount (Margittai et al. 2018a); for example, the potential loss of 100$ will weigh more than the potential gain of 100$ in the decision-making process.

Moreover, “intertemporal decisions” refer to choices that involve outcomes realized at different points in time (Kalenscher and Pennartz 2008). For example, one might choose between withdrawing cash from their bank account to have access to the money right now, or investing it in a fixed-term account, which promises a profit in the future but restricts access to money in the present. In intertemporal choice, participants can be distinguished by their tendency to forego future profits in favor of sooner available assets.

Another type of decision, “decisions with reward anticipation,” has a focus on the valuation of the outcome by the brain reward system. Generally, a positive outcome makes the individual behave in a manner that increases the probability of getting the reward again; this behavior is linked with learning (Knutson et al. 2001, Kirsch et al. 2003). Importantly, though, the brain reward system is not only active when a reward is delivered but also in the anticipation of it.

Psychological research on “moral decision-making” is concerned with describing the regularities and mechanisms of people’s responses to moral dilemmas without claim to normativity. Moral dilemmas can be defined as “a situation in which the decision-maker has to give priority to one moral value over another” (Kvalnes 2015, p. 14). Dilemmas “arise when […] two or more such values conflict in the perception of a decision-maker, or when one is assessing another’s moral choice” (Maclagan 2003, p. 22). In the standard paradigm, participants are presented with two choice options, where one achieves a preferable outcome via problematic means (e.g. sacrificing the few for the many; often dubbed “utilitarian” in loose reference to the eponymous Utilitarian ethics) while the other one refrains from such problematic means to the result of a less preferable outcome (e.g. preserving inalienable rights at all costs; often dubbed “deontological” in loose reference to Kantian ethics).

The domain of social choice deals with decisions that directly involve others. Generally, people tend to exhibit prosocial behavior toward close others. That is, they are willing to forego their benefit for that of others. However, this generosity decreases as a function of social distance. This decrease in generosity with increasing social distance is known as social discounting (Margittai et al. 2018a).

The goal of this systematic review is to enhance our understanding of the causal role of the HPA axis and SAM system in these different types of decision-making.

## Methodology

### Registration

This systematic review adheres to the PRISMA guidelines (Page et al. 2021) and is registered in the PROSPERO database with the number CRD42021211293.

#### Inclusion criteria and search strategy

The research question followed the PICO strategy (Population, Intervention, Comparator, and Outcome). Specifically, the population was healthy adults (P), with the intervention being stress neuromodulators administered pharmacologically (I), the comparator was a placebo or control group (C), and the outcome was decision-making (O). The initial research question aimed to determine whether pharmacologically administered drugs altering the action of stress modulators produce changes in decision-making. It is important to note that the HPA axis and the SAM system do not exclusively nor exhaustively characterize the stress response, as explained in the introduction.

The exclusion criteria included review articles, case studies, and research in animals, book chapters, and letters to the editor, editorials, protocols, pilot studies, and studies that did not adhere to the PICO question guideline. The searched databases were “APA PsycNet,” “Cochrane,” “PubMed,” “ScienceDirect,” “Scopus,” and “Web of Science.” Manual searches, such as “Google Scholar” and checking the reference list of relevant publications, were also performed by the reviewers. For the search strategy, the controlled vocabulary of MESH (Medical Subject Heading) was used, incorporating terms such as “Hydrocortisone, Fludrocortisone, Norepinephrine, Noradrenaline, Cortisol, Glucocorticoids, Decision-making, Temporal Discounting, and Decision-making under risk.” Detailed information on the search strategy is available in the supplementary material.

An alert was programmed in each database to report new studies, and there was no language or time restriction in the search. Two reviewers (L.F.S. and J.A.R.-F.) independently performed the searches, selected the studies, conducted data extraction, and analyzed the risk of bias. The searches were conducted in June 2021, and references were collected in the citation manager Mendeley.

#### Data extraction

We employed Mendeley’s automatic duplicate removal, supplemented by a manual duplicate screening of our dataset. Subsequently, studies underwent title and abstract screening based on pre-established inclusion/exclusion criteria. The remaining studies underwent a full-text review, with those not meeting the inclusion criteria being excluded. Finally, we extracted key information from the included studies, such as authors, study design, sample details (sex, size, and age mean), time between stress onset and decision-making, drug administration specifics, details of the decision-making task, statistical analysis methods, and study results (refer to Table 1 for details).

**Table 1.** Summary and characteristics of the included studies.

		Sample						
Author/year	Design	Participants	Size	Mean Age ± SD (range)	Time between drug administration and decision-making task	Drug administered	Decision-making domain/task	Results
Cueva et al. 2015.	Randomized double-blind placebo within-subject design	Male and female	29	25.7 ± 2.68	60 min	100 mg Hydrocortisone -Placebo	Financial risk taking Trading task: experimental asset market.	Hydrocortisone administration had no effect in overall investment (*p > *0.05). After hydrocortisone administration there was a significant increased mean investment in high variance stock (willingness to take risk) (*P < *.03).
Deuter et al. 2017.	Randomized double-blind placebo between-subject design	Male and female	80; CG: 40; EG:40	CG: 23,8 ± 3.6 EG: 24.1 ± 3.1	150 min	0.4 mg Fludrocortisone—Placebo	Risk taking the BART.	Participants after fludrocortisone administration made more pumps per trial (*p < *.032) and had a higher number of balloon explosions *(p =* .027).
Günthner et al. 2018.	Randomized double-blind placebo between-subject design	Male and female	54; CG:34; EG:20	CG: 22.15± 0.51 EG: 22.10 ± 0.73	N/A	20 mg Hydrocortisone—Placebo	Reward learning (reward anticipation) Computational learning model.	There were found no differences between groups *(p = *.08).
Herman et al. 2019.	Randomized double-blind placebo between-subject design	Male and female	42; CG: 21; EG:21	Placebo: 21.29 ± 3.27 Yohimbine: 23.19 ± 5.41	45 min	20 mg Yohimbine—Placebo	Temporal and Probability discounting Probability discounting task; Monetary choice questionnaire.	There were found no differences between groups (*p = *.315, *p = *.499).
Kinner et al. 2016.	Randomized double-blind placebo between-subject design.	Male and female	60; CG: 30; EG: 30	24.0 ± 3.4	30 min	30 mg Hydrocortisone—Placebo	Reward anticipation Monetary Incentive Delay Task.	There were found no differences between groups (p > .1).
Kluen et al. 2017.	Randomized double-blind placebo between-subject design	Male and female	103; CG: 27; Cortisol group: 25	24.79 ± 0.36	85 min	20 mg Hydrocortisone 20 mg Yohimbine 20 mg Hydrocortisone + 20 mg Yohimbine Placebo	Risk taking. BART.	Participants after hydrocortisone administration (with or without Yohimbine) exploded a higher number of balloons (*p = .003*).
Lempert et al. 2012.	Non-randomized double-blind placebo within-subject design.	Male and Female	37	27.8 ± 6.6	90 min	80 mg Propranolol—Placebo	Intertemporal choice task (temporal discounting).	There were found no differences between groups *p = .434*.
Margittai et al. 2018a.	Randomized double-blind placebo between-subject design	Male	N: 92; Control Group (n: 24); Placebo + Yohimbine (n: 21); Placebo + Hydrocortisone (n: 24); Yohimbine + Hydrocortisone (n = 23)	CG: 26.62 ± 7.24 Yohimbine 22.85 ± 3.00 Hydrocortisone 25 ± 5.89 Yohimbine + Hydrocortisone 24.29 ± 8.96	45 min	20 mg Hydrocortisone 20 mg Yohimbine 20 mg Hydrocortisone + 20 mg Yohimbine Placebo.	Risk taking/loss aversion risk and loss aversion task.	Risk aversion was not affected. Loss aversion in participants who received both drugs was significantly lower (*p *≤ .018).
Metz et al. 2020.	Randomized double-blind placebo between-subject design	Male	N: 104; Control Group (n: 26); Placebo + Yohimbine (n: 26); Placebo + Hydrocortisone (n: 26); Yohimbine + Hydrocortisone (n: 26)	CG: 23.81 ± 3.36 Placebo + Yohimbine 23.19 ± 3.29 Placebo + Hydrocortisone 24.54 ± 4.04 Yohimbine + Hydrocortisone (M: 2469 ± SD: 3,44)	Yohimbine: 60 min; Hydrocortisone: 45 min	10 mg Hydrocortisone 10 mg Yohimbine 10 mg Hydrocortisone + 10 mg Yohimbine Placebo	Risk taking/loss aversion risk and loss aversion task.	Participants after hydrocortisone administration (with or without yohimbine) had significantly lower choice frequencies in the gain-only trials (p < .01) but not in the mixed-gamble trials (p < .160). No effect of hydrocortisone (p < .55) or yohimbine (p < .09) or both (p < .30) on loss aversion.
Montoya et al. 2014.	Randomized double-blind within-subject design	Male	20	23 ± 3.4	40 min	40 mg Hydrocortisone—Placebo	Reward anticipation Monetary Incentive Delay Task.	There were no differences between groups (*p *≤ .217).
O’Carroll and Papps 2003.	Randomized double-blind placebo between-subject design	Male and female	N: 30; Control Group (n: 11) Experimental group 8 mg (n: 11) Experimental group 4 mg (n: 8)	CG 20.0 ± SD =3.1 8 mg Reboxetine 21.8 ± 4.6 4 mg Reboxetine 19.3 ± 0.9	120 min	8 mg Reboxetine 4 mg Reboxetine Placebo	Risk taking Iowa Gambling Task.	There were no differences between groups (*p *= 1.00).
Putman et al. 2010.	Non-randomized double-blind placebo within-subject design	Male	N = 29; Control group (n:15). Experimental group (n: 14)	22.7 ± 2.5	120 min	40 mg Hydrocortisone—Placebo	Risk taking The Gambling monetary task (binary decisions).	Subjects made more high-risk experimental games after hydrocortisone administration. Drug condition significantly influenced the probability of losing contrast score (p < .05).
Riis-Vestergaard et al. 2018.	Randomized double-blind placebo between-subject design	Male	78	22.26 ± 0.70	Rapid cohort: 15 min Slow cohort: 195 min	10 mg Hydrocortisone—Placebo	Temporal discounting intertemporal choice task	Participants after hydrocortisone administration showed a strongly increased preference for the small, soon reward over the larger, delayed reward. This effect was not found when testing occurred 195 min after hydrocortisone administration (*p *> .05).
Robertson et al. 2016.	Randomized double-blind placebo within-subject design	Male	11	39.6 ± 10.4	60 min	50 mg Cortisone—Placebo	Risk taking BART	There were no differences between groups.
Rogers et al. 2004.	Non-randomized double-blind placebo between-subject design	Male and female	32	Males: 23.59 ± 2.01 Females: 20.07 ± 0.81	75 min	80 mg Propranolol—Placebo	Decision-Making Task (Gambling task)	It significantly attenuated volunteers’ discrimination between the magnitude of possible losses in situations where the probability of winning was relatively low, and the probability of suffering losses was relatively high p < .05
Sokol-Hessner et al. 2015.	Non-randomized double-blind placebo within-subject design	Male and female	47	26.6 ± 5,1	90 min	80 mg Propranolol -Placebo	Gambling Task	There were no differences between groups (*P* = .34).

Note. CG: Control Group; EG: Experimental Group; SD: Standard Deviatio.

#### Data analysis: risk of bias

We employed the Rob 2.0 software (Sterne et al. 2019) to assess the risk of bias in randomized studies and the ROBINS I software (Sterne et al. 2016) for non-randomized studies. In the case of between-subject-design studies, we utilized the standard RoB 2 version, while for within-subject-design studies, we employed the RoB 2 tool designed for crossover trials. These tools consist of questions that help assess various design, conduct, and reporting aspects of the studies. Our focus centered on specific domains: Missing outcome data, measurement of the outcome, and selection of the reported results. Responses to these questions were recorded on a 4-point Likert scale (“Yes,” “Probably Yes,” “Probably No,” or “No”). In the risk of bias analysis, a domain is categorized as “high” if there is a high risk of bias, “some concerns” if there are minor problems, or “low” if there is a low risk of bias.

## Results

### Selection of the studies

**Figure 1.**
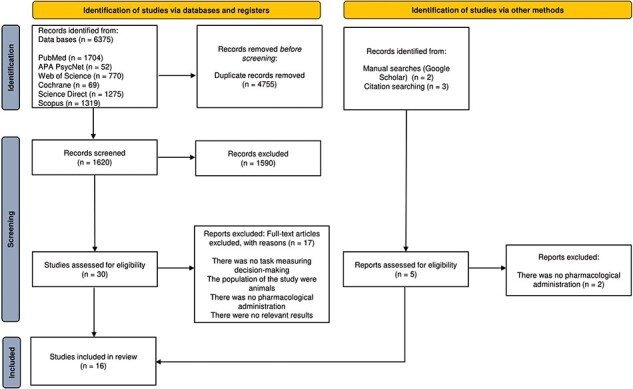
Flow diagram.

*Note*. Adapted PRISMA flow diagram of the study selection.

In total, we identified 6375 studies through our database searches (4755 studies after removing duplicates). Title and abstract screening led to the selection of 30 studies for full-text reading. Ultimately, 16 studies met the inclusion criteria for the systematic review (see Table 1) based on our full-text evaluation (refer to Fig. 1). Additionally, three studies met the inclusion criteria but focused on specific decision types for which no sufficient research has been conducted for a meaningful review, i.e. as moral decisions (Sylvia et al. 2013), social discounting (Margittai et al. 2018b), and approach-avoidance decisions (van Peer et al. 2007). Notably, these studies did not enter the systematic review, as they were the only representatives for the decision type they studied. However, for completeness, we mention some key findings: cortisol enhanced congruency effects for angry faces in highly avoidant individuals (van Peer et al. 2007), propranolol increased judgments of harmful actions as morally unacceptable (Sylvia et al. 2013), and hydrocortisone administration promoted prosocial tendencies toward close others (Margittai et al. 2018b). We will not discuss them further, acknowledging that our review does not address these specific types of decision-making (see Limitations).

### Risk of bias analysis

The risk of bias was assessed using the RoB 2 tool for randomized studies and the ROBINS I for non-randomized studies. The domains evaluated included the randomization process, missing outcome data, measurement of the outcome, and selection of the reported results. Additionally, for within-design studies, we assessed the domain of bias arising from period and carryover effects (see Supplementary Figs S1 and S2).

Nine studies were evaluated with the RoB 2 tool for parallel trials (O’Carroll and Papps 2003, Kinner et al. 2016; Deuter et al. 2017, Kluen et al. 2017, Günthner et al. 2018, Riis-Vestergaard et al. 2018, Margittai et al. 2018a, Herman et al. 2019, Metz et al. 2020), and three with RoB 2 for crossover trials (Montoya et al. 2014, Cueva et al. 2015, Robertson et al. 2016). All 12 studies were assessed as having a low risk of bias. Furthermore, four studies were evaluated with the ROBINS I tool (Rogers et al. 2004, Putman et al. 2010, Lempert et al. 2012, Sokol-Hessner et al. 2015).

#### Characteristics of the studies

Out of the 16 studies, 12 employed a randomized design, with 9 using a between-subject design (O’Carroll and Papps 2003, Kinner et al. 2016, Deuter et al. 2017, Kluen et al. 2017, Günthner et al. 2018, Riis-Vestergaard et al. 2018, Margittai et al. 2018a, Herman et al. 2019, Metz et al. 2020) and 3 a within-subject design (Montoya et al. 2014, Cueva et al. 2015, Robertson et al. 2016). The remaining four studies utilized a non-randomized design, with three employing a within-subject design (Putman et al. 2010, Lempert et al. 2012, Sokol-Hessner et al. 2015) and one a between-subject design (Rogers et al. 2004).

Of the 16 studies, 6 included only male participants, while 10 included both male and female participants. No studies exclusively included female participants. Regarding drug (stress neuromodulator) administration, three studies used hydrocortisone and yohimbine (Kluen et al. 2017, Margittai et al. 2018a, Metz et al. 2020), seven studies used only hydrocortisone (Putman et al. 2010, Montoya et al. 2014, Cueva et al. 2015, Kinner et al. 2016, Robertson et al. 2016, Günthner et al. 2018, Riis-Vestergaard et al. 2018)—the study by Robertson et al. (2016) used exogenous cortisol, cortisone, which is converted in the liver to hydrocortisone—, one study utilized fludrocortisone (Deuter et al. 2017), one used reboxetine (O’Carroll and Papps 2003), one used only yohimbine (Herman et al. 2019), and three used propranolol (Rogers et al. 2004, Lempert et al. 2012, Sokol-Hessner et al. 2015) [See Box 1 for an overview of the drugs and their pharmacology].

The included studies explored five different domains of decision-making as outlined above (refer also to Box 2). Specifically, eight studies focused on measuring risk-taking (O’Carroll and Papps 2003, Putman et al. 2010, Cueva et al. 2015, Robertson et al. 2016, Deuter et al. 2017, Kluen et al. 2017, Margittai et al. 2018a, Metz et al. 2020). Among these, two studies also assessed loss aversion (Margittai et al. 2018a, Metz et al. 2020). Additionally, Rogers et al. (2004) and Sokol-Hessner et al. (2015) exclusively measured loss aversion, bringing the total number of studies assessing loss aversion to four. Furthermore, three studies delved into temporal discounting (Lempert et al. 2012, Riis-Vestergaard et al. 2018, Herman et al. 2019), while three studies investigated reward anticipation (Montoya et al. 2014, Kinner et al. 2016, Günthner et al. 2018).

We opted against conducting a quantitative synthesis of the data (i.e. a meta-analysis) due to the heterogeneity in the methodology across studies. Specifically, variations in the outcome measures and the diverse action mechanisms of the drugs used, coupled with the limited number of studies included, led us to this decision (see Discussion).

#### Drugs acting upon the HPA axis

The most extensively investigated domain of decision-making using pharmacological interventions targeting the HPA axis is decisions under risk. Despite the relatively high number of investigations, the current evidence presents an apparently inconsistent picture regarding HPA axis modulation.

On the one hand, the administration of 100 mg of hydrocortisone resulted in an increased investment in a financial risk-taking task (Cueva et al. 2015), and after the administration of 40 mg of hydrocortisone, subjects chose riskier gambles (Putman et al. 2010). Another study observed that after the administration of 20 mg of fludrocortisone, participants made more pumps per trial and had a higher number of explosions in the Balloon Analog Risk Task (BART) (Deuter et al. 2017). Furthermore, after the administration of 20 mg of hydrocortisone and 20 mg of yohimbine (but not yohimbine alone), participants exhibited more exploded balloons in the BART, indicating increased risk-taking (Kluen et al. 2017).

On the other hand, in contrast, other studies found no changes in the BART after the administration of 50 mg of cortisone (Robertson et al. 2016), and there was no effect on another risk-taking task after the administration of either 20 mg of hydrocortisone or 20 mg of yohimbine, or after the administration of both drugs combined (Margittai et al. 2018a). Finally, one study reported the reverse result: after the administration of 10 mg of hydrocortisone combined or not with 10 mg of yohimbine, there was less risk-seeking in gain-only trials (Metz et al. 2020).

**Figure 2.**
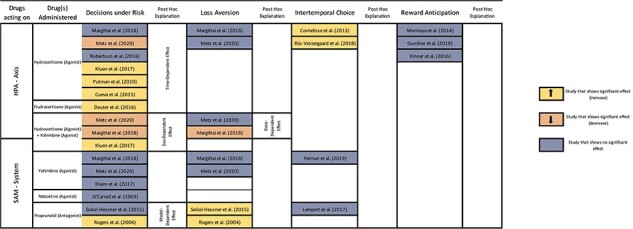
Graphical overview of the evidence for drug effects across decision-making domains.

*Note*. Drugs, decision-making task, its significant effects and the post-hoc explanation.

In other choice domains, there is a more limited number of studies at this point. In the domain of losses, evidence is similarly ambiguous for HPA axis modulators, as for decisions under risk in the domain of gains. One study found that the administration of 20 mg of hydrocortisone combined with 20 mg of yohimbine reduced loss aversion, but neither drug alone had this effect (Margittai et al. 2018a). Another study, however, found no effect of 10 mg of hydrocortisone, 10 mg of yohimbine, combined or alone (Metz et al. 2020). In temporal discounting Riis-Vestergaard et al. (2018) found that after the administration of 10 mg of hydrocortisone, participants tested 15 min later exhibited a strong preference for the smaller and sooner reward. However, this effect was not present in groups tested 195 min later. Several studies, including those by Günthner et al. (2018) with 20 mg, Kinner et al. (2016) with 30 mg, and Montoya et al. (2014) with 40 mg of hydrocortisone, did not observe any effect on reward anticipation [see Fig. 2].

### Drugs acting upon the sympathetic–adrenal–medullary system

For decision-making under risk, yohimbine alone did not show any effect (Metz et al. 2020). Another study reported that the administration of neither 4 mg nor 8 mg of reboxetine had any impact on the Iowa Gambling Task (O’Carroll and Papps 2003). Additionally, Sokol-Hessner et al. (2015) found no effect after the administration of 80 mg of propranolol.

Regarding loss aversion, two studies administered propranolol with different results. The administration of 80 mg of propranolol reduced the discrimination between large and small possible losses when the probability of winning was relatively low and the probability of losing was high in one study (Rogers et al. 2004). Conversely, Sokol-Hessner et al. (2015) found no effect on loss aversion after administering 80 mg of propranolol. However, *post hoc* analysis revealed a dose-dependent reduction in loss aversion. For temporal discounting, neither the administration of 20 mg of yohimbine (Herman et al. 2019) nor 80 mg of propranolol had any effect (Lempert et al. 2012).

Box 1. Drugs that act on HPA-axis and SAM-system.

**Table:** 

Drug	Pharmacodynamics	Used for
Hydrocortisone	Hydrocortisone, or cortisol, is a glucocorticoid secreted by the adrenal cortex. It is essential for life and supports many important cardiovascular, metabolic, immunologic, and homeostatic functions. Corticosteroids usually are metabolized in the liver and excreted by the kidney. It binds to glucocorticoid receptors and mineralocorticoid receptors. The mechanism of action of hydrocortisone is as a corticosteroid hormone receptor agonist.	Used to treat corticosteroid-responsive dermatoses, endocrine disorders, immune and hematologic conditions, inflammation, and allergic disorders.
Fludrocortisone	Fludrocortisone is a synthetic adrenal steroid with high mineralocorticoid activity; it is typically used to replace endogenous aldosterone—the main mineralocorticoid steroid hormone—in some medical conditions. Its mechanism of actions on alpha adrenoreceptors are like endogenous mineralocorticoid. It is metabolized by the liver. Fludrocortisone also acts on glucocorticoid receptors but with lower affinity.	Used in the treatment of adrenocortical insufficiency associated with mineralocorticoid deficiency, and salt-losing adrenogenital syndrome. It also has anti-inflammatory and immunosuppressive effects.
Yohimbine	Yohimbine blocks alpha-2 adrenergic autoreceptors, thus increasing noradrenaline release into the synaptic cleft. It produces central nervous system (CNS) stimulation, and sympathetic activation increasing heart rate and blood pressure. It is typically used in erectile dysfunction. The effects of yohimbine in erectile ability may be due to the increase in norepinephrine release and in firing rate of neurons of the LC. Also, in high concentrations yohimbine may interact with dopamine, serotonin, and alpha-1 adrenergic receptors.	Used in the treatment of erectile dysfunction.
Reboxetine	Reboxetine is a selective noradrenaline reuptake inhibitor, a new antidepressant class. Reboxetine acts by binding to the norepinephrine transporter and blocking reuptake of extracellular norepinephrine. Consequently, it increases of noradrenaline availability in the synaptic cleft and modification of noradrenergic transmission. Reboxetine does not affect dopamine or serotonin reuptake. It has low affinity for adrenergic, cholinergic, histaminergic, dopaminergic, and serotonergic receptors.	Used for the treatment of depression.
Propranolol	Propranolol is a non-selective beta-adrenergic receptor antagonist. It competes with sympathomimetic neurotransmitters for binding to receptors, which inhibits sympathetic stimulation of the heart. Beta-adrenergic blocking agents are medications that reduce blood pressure. It reduces resting heart rate, cardiac output, blood pressure. Propranolol blocks the action of endogenous catecholamines, epinephrine, and norepinephrine at beta adrenoceptors.	Used to treat hypertension, angina, atrial fibrillation, myocardial infarction, migraine, essential tremor, hypertrophic subaortic stenosis, and pheochromocytoma.
Dexamethasone	Dexamethasone is an artificial corticosteroid. It is an agonist of the glucocorticoid receptor and is highly selective of glucocorticoid receptor over the mineralocorticoid receptor. It is used for the treatment of various inflammatory conditions, and administration of dexamethasone results in a dose-dependent suppression of the HPA axis.	Used for the treatment of inflammatory conditions, such as bronchial asthma, and endocrine and rheumatic disorders.
Spironolactone	Spironolactone is a specific pharmacologic antagonist of aldosterone—a main mineralocorticoid hormone. Competitively inhibits mineralocorticoid receptors by blocking the mineralocorticoid receptor, the spironolactone inhibits the effects of mineralocorticoids in the body and increase levels of aldosterone.	Used for the treatment of hypertension, hyperaldosteronism, edema due to various conditions, hirsutism, and hypokalemia.

Note. Pharmacodynamics of each drug used, and the use related to medical condition; From “Drugbank online database”, by Wishart et al. (2006).

Box 2. Stress Neuromodulators drug used.

**Table:** 

Drug	Properties	Expected Action
Hydrocortisone (Cortisol)	Cortisol is a glucocorticoid secreted by the adrenal cortex. It is a corticosteroid hormone receptor agonist.	Acts on major stress neuromodulator cortisol (stimulating). Emulates the cortisol stress response.
Fludrocortisone	Fludrocortisone is a synthetic corticosteroid, and a mineralocorticoid agonist.	Leads to mineralocorticoid receptor stimulation. Emulates the cortisol stress response specifically in the mineralocorticoid receptor.
Yohimbine	Yohimbine blocks presynaptic alpha-2 adrenergic receptors.	Leads to increased noradrenaline release. Increases arousal, heart rate and is used to emulate the noradrenergic stress response.
Reboxetine	Reboxetine is a selective noradrenergic reuptake inhibitor. It is a drug that blocks the transport of adrenergic transmitters into axon terminals.	Manipulates the central noradrenergic system increasing noradrenaline availability in the synaptic cleft. Acts to amplify the somatic marker (the emotional reaction from the autonomic nervous system).
Propranolol	Propranolol is a synthetic, non-selective beta-adrenergic receptor blocker.	Suppresses noradrenergic activation by blocking beta 1 and beta 2 adrenoceptors and reduces physiological markers of high arousal (e.g. emotional arousal).

Note. Properties of each drug used, and the expected effect related to stress.

Box 3. Decision tasks.

**Table:** 

Task	Description	Study
Intertemporal choice task	Participants have to make decisions between a sooner smaller reward and a later larger reward.	Herman et al. (2019); Lempert et al. (2012); Riis-Vestergaard et al. (2018).
Trading task: Experimental asset market	Participants play an asset trading game that mimics the key features of a real-world financial market.	Cueva et al. (2015).
BART	Participants saw one balloon at a time on a computer screen and were instructed that the goal of the task was to win money by pumping up the balloons without exploding.	Deuter et al. (2017); Kluen et al. (2017); Robertson et al. (2016).
Monetary Incentive Delay Task	There were three experimental conditions: S−(control), vS+ (verbal reward) and mS+ (monetary reward). Participants had to respond as fast as possible to a bright flashlight following the presentation of the vS+ and the mS+ by pressing a response button.	Günthner et al. (2018); Kinner et al. (2016); Montoya et al. (2014).
Risk and loss aversion task	Participants made binary choices between receiving amount (x) for sure and a lottery, where they had a probability of either winning amount (y) or losing amount (z). Choice options were dynamically selected based on participants’ prior answers, according to an informational criterion that optimized the estimation of individual parameters describing loss aversion, and risk aversion.	Margittai et al. (2018a); Metz et al. (2020).
Iowa Gambling Task	Participants play a card game where the player is instructed to win as much money as possible over 100 selections from 1 of 4 decks. The rules are not disclosed. Two of the decks are “high risk”—that is, intermittently produce large rewards but in the long-term lead to significant financial losses, whereas two decks lead to modest but consistent gains.	O’Carroll and Papps (2003).
Gambling Monetary Task	This task contains trials in which subjects have to choose between two gambles with the aim of earning as many as possible.	Putman et al. (2010); Rogers et al. (2004); Sokol-Hessner et al. (2015).

Note. Descriptive summary of the task used to measure decision-making.

## Discussion

The overarching goal of this systematic review is to enhance our understanding of the impact of the HPA axis and SAM system on decision-making. An additional objective is to utilize the more controlled and specific nature of pharmacological manipulations to bring clarity to the sometimes-inconsistent research on decision-making using behavioral stress protocols (see Box 3). However, it is crucial to note that the administration of drugs affecting the HPA axis and/or SAM systems differs from the acute stress response, a distinction sometimes overlooked in the literature (Schwager et al. 2014, Schippers et al. 2016). Our consistent findings are as follows: Isolated SAM system stimulation did not affect risk aversion, loss aversion, or intertemporal choice. Isolated SAM system inhibition, tentatively, reduced sensitivity for losses but did not affect risk aversion or intertemporal choice. However, for several decision-domain × drug-type combinations, results are less clear.

Our discussion is, therefore, organized into a summary of results for each decision domain, outlining consistent and inconsistent findings, and comparing pharmacological results with acute stress findings. Additionally, we adopt a theory-driven approach to explain inconsistencies, evaluating alternative post hoc explanations to the null hypothesis that inconsistencies are random statistical artifacts (i.e. alpha or beta errors). Specifically, these alternative explanations include dose-dependency effects, differential receptor stimulation of drugs, timeline effects (especially differential involvement of genomic and non-genomic cortisol effects), inclusion or exclusion of female participants, and substantial differences in statistical power. In the domain of decisions under risk, we explore whether the task involved explicit risk or ambiguity.

### Decision under risk

Consistently, the administration of SAM system stimulants, namely yohimbine (Kluen et al. 2017, Margittai et al. 2018a, Metz et al. 2020) and reboxetine (O’Carroll and Papps 2003), did not impact decisions under risk. Consequently, a uniform increase in SAM system activity beyond baseline levels, as expected early after stress exposure, does not appear to influence risk-taking. This suggests that the previously observed effects of acute stress on risk-taking (such as riskier and less advantageous choices; Starcke and Brand 2016) cannot be solely explained by the activation of the SAM system alone. This finding also counters the notion in some literature that absolute arousal levels uniformly influence choices under risk (e.g. Jahedi et al. 2017, who found a small positive effect). However, it is premature to definitively conclude that sympathetic activity is not involved in decision-making, as the administration of the SAM system inhibitor propranolol produced some results (Rogers et al. 2004, Sokol-Hessner et al. 2015). For instance, it is plausible that sympathetic arousal serves as a physiological signal to evaluate risky prospects, but this comparative process may not be strongly dependent on the absolute level of arousal (see e.g. Studer et al. 2016). Or the action of sympathetic arousal is aligned to weighting of the losses specifically (Rogers et al. 2004, Sokol-Hessner et al. 2015).

As noted earlier, the results on decision-making under risk after the administration of propranolol are not entirely null (see Fig. 2). Studies were comparable across key methodological dimensions: both utilized a similar task, included a mixed-gender sample, and had comparable sample sizes (descriptively larger for the null finding). Experimental timelines were slightly different: Rogers et al. (2004) had a 75-min drug–administration–task delay, whereas Sokol-Hessner et al. (2015) had a 90-min administration–task delay. However, Sokol-Hessner *et al*. argue that the divergent results stem from Rogers *et al*. failing to differentiate the contributions of loss aversion, risk attitudes, and choice consistency. If we follow this argument, an effect of propranolol on decisions under risk is likely to be small and likely restricted to the weighing of the losses.

For modulators of the HPA axis, results appear inconsistent as well (see Fig. 2). However, these seemingly inconsistent outcomes for decisions under risk after stimulation of the HPA axis could tentatively be explained by varying time delays between drug and task administration among studies. Risk-taking increased after 60 min (Cueva et al. 2015), 85 min (Kluen et al. 2017), 120 min (Putman et al. 2010), and 150 min (Deuter et al. 2017), whereas after 45 min (Margittai et al. 2018a) or 60 min (Robertson et al. 2016), risk-taking was unaffected or even decreased (Metz et al. 2020; 45 min). A positive effect on risk-taking seems to manifest only at least 60 min after drug administration, aligning temporally with the possible onset of genomic cortisol effects. Therefore, the differential presence of a genomic glucocorticoid effect could explain the inconsistencies in the results. Note that this is not aligned, however, with a recent meta-analysis comparing risky decisions of stressed vs. non-stressed participants (Starcke and Brand 2016). The meta-analysis found that in stress conditions, participants made more risky decisions but explicitly rejected the hypothesis of a time effect. This underscores that natural stressors cannot be reduced to the activation of the HPA axis.

Finally, results for decisions under risk after the administration of hydrocortisone plus yohimbine also appear inconsistent (see Fig. 2). Here, the divergent results could tentatively be explained by the differential inclusion or exclusion of female participants across studies. Specifically, Kluen et al. (2017) found a positive effect of hydrocortisone plus yohimbine on risk-taking in a mixed sample, whereas Margittai et al. (2018a) and Metz et al. (2020) found no or a negative effect, respectively, in male-only samples. However, the direction of this supposed sex modulation contradicts previous propositions on sex-modulated stress effects that suggested an increase in risk-taking specifically for male participants (Mather and Lighthall 2012).

### Loss aversion

In contrast to risk-taking in the gain domain, Rogers et al. (2004) observed a decrease in risk-taking involving losses (a decrease in loss aversion) following the administration of propranolol. On the other hand, Sokol-Hessner et al. (2015) did not find any direct effect on risk-taking, although they observed a similar effect when considering dose-dependency. While a detailed comparison of results is challenging due to differing analytical approaches, both studies imply that propranolol reduces sensitivity to the magnitude of losses. Additionally, there are consistent null findings of hydrocortisone administration alone on decision-making involving losses (Margittai et al. 2018a, Metz et al. 2020), which is in line with results from studies using psychosocial stressors (Sokol-Hessner et al. 2016). Lastly, results after the administration of hydrocortisone plus yohimbine are inconsistent but could be quite straightforwardly explained by the dose used. Metz et al. (2020) used a lower dose, 10 mg, and did not find an effect, while Margittai et al. (2018a) used 20 mg and found a significant negative effect. This might also be helpful for the interpretation of null findings under psychosocial stress (Sokol-Hessner et al. 2016); perhaps, loss aversion is only affected at very high levels of stress.

### Intertemporal decisions

Interestingly, two studies that modulated the SAM system using yohimbine (Herman et al. 2019) or propranolol (Lempert et al. 2012) found no effect, tentatively suggesting that the noradrenergic component of the stress response is less relevant for intertemporal decision-making. HPA axis stimulation (Riis-Vestergaard et al. 2018) influenced intertemporal choices in one study. Specifically, the reviewed studies indicated an increased preference for the smaller, and sooner reward when tested 15 min after the administration of HPA axis stimulants but not when tested later. The timeline interaction of HPA axis effects is consistent with our theoretical knowledge about the temporal dynamics of neuronal corticosteroid effects (Hermans et al. 2014), where later genomic cortisol effects might reverse early non-genomic effects. Furthermore, the results align with other studies on intertemporal decisions where psychosocial stress was induced using the TSST: similarly, in these studies, stress increased choices for the smaller and sooner reward shortly after stress induction (i.e. before the onset of genomic cortisol effects) (Kimura et al. 2013, Haushofer et al. 2021). Moreover, a meta-analysis showed a positive effect size in stressed participants exhibiting higher discounting (Fields et al. 2014; Haushofer et al. 2021). Therefore, the current evidence tentatively highlights the importance of the temporal dimension of the stress response (and especially the HPA axis component). Hence, future studies should carefully calibrate their experimental timeline.

### Reward anticipation

There are consistent null findings for hydrocortisone administration on reward anticipation (Montoya et al. 2014, Kinner et al. 2016, Günthner et al. 2018). This aligns with the results of non-pharmacological stress. Porcelli et al. (2008; 2012) did not find significant differences in reward processing after exposing participants to acute environmental stress, and Oei et al. (2014) did not observe an effect after exposure to acute psychosocial stress.

## Conclusions

Isolated SAM system stimulation yielded no significant impact on risk aversion, loss aversion, or intertemporal choice. Conversely, SAM system inhibition exhibited a tentative reduction in sensitivity to losses without affecting risk aversion or intertemporal choice. Theoretical considerations suggest that the timing between drug and task administration may not significantly moderate the effects of isolated SAM system stimulation, provided that task administration aligns with drug-specific pharmacokinetics (see Box 1), given the predominantly immediate effects of catecholamines.

Furthermore, isolated HPA axis stimulation demonstrated no discernible effects on loss aversion or reward anticipation. Notably, time-dependent effects may explain the variability in HPA axis stimulation’s influence on decision-making under risk, with late, genomic effects potentially playing a crucial role. Further research is warranted to confirm this hypothesis.

Finally, combined HPA axis and SAM system stimulation produced inconsistent results which, however, can plausibly be explained by dose differences (loss aversion) and sex differences (risk aversion). Again, these explanations should be confirmed in future research. Another aspect to consider is the potential time-dependency of HPA axis and SAM system interactions. Catecholamines exert immediate effects, contrasting with the combination of rapid non-genomic and slower genomic effects exhibited by corticosteroids. Additionally, different drugs stimulate peak hormone levels at different time points (e.g. hydrocortisone after approximately 60 min and Yohimbine after 30–60 min; see Box 1). Hence, drug administration must be carefully timed to understand the nature of potential interaction effects.

## Limitations

While our systematic review followed a pre-registered strategy, adhered to recommended guidelines, and employed theory-based interpretation of the results, we acknowledge some limitations and caveats in interpreting our findings. Primarily, it should be noted that the body of included studies is limited both in size and scope. This means that many combinations of decision domains and drugs have not been extensively studied yet. For the interpretation of our review, this implies, first, that our results do not encompass certain decision-making domains such as social or moral choices. Second, given the limited number of studies per cell (see Fig. 2), we opted against a quantitative assessment of the evidence, deviating from our initial registration. This inevitably introduces a limitation to the confidence of our conclusions.

Therefore, we view the results of our review as a starting point for future research: identifying areas that have received limited attention in the literature and pointing out potential inconsistencies. Additionally, we propose theory-based *post hoc* explanations that can be tested in future confirmatory studies.

## Supplementary Material

nsae069_Supp
