# Correction to: Do stress hormones influence choice? A systematic review of pharmacological interventions on the HPA axis and/or SAM system

**DOI:** 10.1093/scan/nsag055

**Published:** 2026-07-24

This is a correction to: Luis Felipe Sarmiento, Jorge Alexander Ríos-Flórez, Fabio Alexis Rincón Uribe, Rafael Rodrigues Lima, Tobias Kalenscher, Amauri Gouveia, Jr, Felix Jan Nitsch, Do stress hormones influence choice? A systematic review of pharmacological interventions on the HPA axis and/or SAM system, *Social Cognitive and Affective Neuroscience*, Volume 19, Issue 1, 2024, nsae069, https://doi.org/10.1093/scan/nsae069

The wrong Figure 2 was presented in the original publication of the article. This should read:

**Figure:**
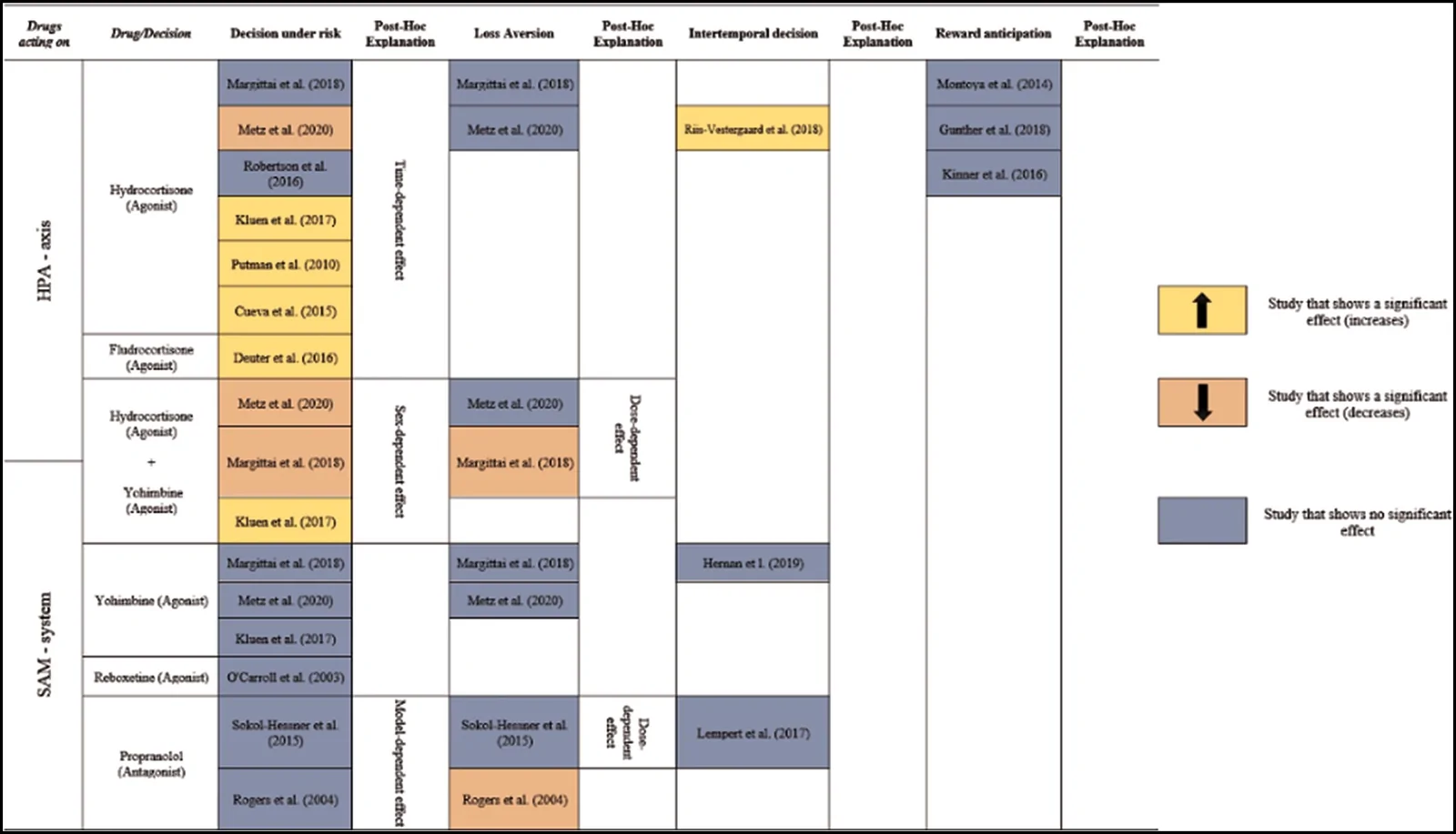


Additionally, the originally published boxes **1-3** should have been a revised **Box 1** and **Box 2** only. **Box 1** should therefore read as follows:

The originally published **Box 2** should be disregarded.

The originally published **Box 3** should be headed and considered as **Box 2,** while in the body of the text the published reference “(refer also to Box 2)” should be disregarded; and published reference “(see Box 3)” should be considered instead to read: “(see Box 2)”.

The errors are outlined only in this correction notice to preserve the version of record.

**Box 1.** *Drugs that act on HPA-axis and SAM-system*.

Drug	Pharmacodynamics	Pharmacokinetics	Used for
Hydrocortisone	Cortisol is a glucocorticoid naturally secreted by the adrenal cortex and functions as a Corticosteroid Hormone Receptor Agonist. Hydrocortisone, its synthetic counterpart, is used as medication in certain medical conditions. It is essential for supporting cardiovascular, metabolic, immunologic, and homeostatic functions. Hydrocortisone binds to glucocorticoid receptors and mineralocorticoid receptors, acting as a Corticosteroid Hormone Receptor Agonist. Typically metabolized in the liver and excreted by the kidneys.	Hydrocortisone is primarily absorbed in the small intestine and subsequently metabolized in the liver. Peak blood concentration occurs approximately 1 hour after administration. The drug strongly binds to plasma proteins and is primarily excreted by the kidneys. Its half-life varies but is generally short, around 1.5 to 2 hours.	Used to treat corticosteroid-responsive dermatoses, endocrine disorders, immune and hematologic conditions, inflammation, and allergic disorders.
Fludrocortisone	Fludrocortisone is a synthetic adrenal steroid with high mineralocorticoid activity; it is typically used to replace endogenous aldosterone – the main mineralocorticoid steroid hormone – in some medical conditions. Its mechanism of actions on alpha adrenoreceptors is comparable to endogenous mineralocorticoids. It is metabolized by the liver. Fludrocortisone also acts on glucocorticoid receptors but with lower affinity.	Fludrocortisone is primarily absorbed in the gastrointestinal tract and exhibits a prolonged half-life. It extensively binds to plasma proteins and is mainly metabolized in the liver before being excreted by the kidneys. The time to reach peak blood concentration varies but is typically around 1 to 2 hours after administration.	Used in the treatment of adrenocortical insufficiency associated with mineralocorticoid deficiency, and salt-losing adrenogenital syndrome. It also has anti-inflammatory and immunosuppressive effects.
Yohimbine	Yohimbine blocks alpha-2 adrenergic autoreceptors, thus increasing noradrenaline release into the synaptic cleft. It produces Central Nervous System (CNS) stimulation, and sympathetic activation increasing heart rate and blood pressure. It is typically used in erectile dysfunction. The effects of Yohimbine in erectile ability may be due to the increase in norepinephrine release and in firing rate of neurons of the locus coeruleus. Also, in high concentrations Yohimbine may interact with dopamine, serotonin, and alpha-1 adrenergic receptors.	Yohimbine is rapidly absorbed in the gastrointestinal tract and reaches peak plasma concentration approximately 0.5 to 1 hour after administration. It undergoes extensive metabolism in the liver and has a short half-life, typically around 0.5 to 3 hours. Elimination primarily occurs through urine.	Used in the treatment of erectile dysfunction.
Reboxetine	Reboxetine is a selective noradrenaline reuptake inhibitor, a new antidepressant class. Reboxetine acts by binding to the norepinephrine transporter and blocking reuptake of extracellular norepinephrine. Consequently, it increases noradrenaline availability in the synaptic cleft and noradrenergic transmission. Reboxetine does not affect dopamine or serotonin reuptake. It has low affinity for adrenergic, cholinergic, histaminergic, dopaminergic, and serotonergic receptors.	Reboxetine is well absorbed after oral administration, reaching peak plasma concentration in approximately 2 to 3 hours. It undergoes metabolism in the liver and is primarily excreted in the urine. The elimination half-life varies, typically ranging between 10 to 12 hours.	Used for the treatment of depression.
Propranolol	Propranolol is a non-selective beta-adrenergic receptor antagonist. It competes with sympathomimetic neurotransmitters for binding to receptors, which inhibits sympathetic stimulation of the heart. Beta-adrenergic blocking agents are medications that reduce blood pressure. It reduces resting heart rate, cardiac output, blood pressure. Propranolol blocks the action of endogenous catecholamines, epinephrine and norepinephrine at beta adrenoceptors.	Propranolol is rapidly absorbed after oral administration, reaching its peak plasma concentration in about 1 to 2 hours. It undergoes extensive hepatic metabolism and has an average elimination half-life of approximately 4 hours. The main route of excretion is through urine.	Used to treat hypertension, angina, atrial fibrillation, myocardial infarction, migraine, essential tremor, hypertrophic subaortic stenosis, and pheochromocytoma.
Dexamethasone	Dexamethasone is an artificial corticosteroid. It is an agonist of the glucocorticoid receptor and is highly selective of glucocorticoid receptor over the mineralocorticoid receptor. It is used for the treatment of various inflammatory conditions, and administration of dexamethasone results in a dose-dependent suppression of the HPA axis.	Dexamethasone is rapidly absorbed after oral administration, reaching its peak plasma concentration in about 1 to 2 hours. It has a relatively long elimination half-life, typically ranging from 36 to 54 hours. Dexamethasone is metabolized in the liver and is primarily excreted by the kidneys.	Used for the treatment of inflammatory conditions, such as bronchial asthma, and endocrine and rheumatic disorders.
Spironolactone	Spironolactone is a specific pharmacologic antagonist of aldosterone -a main mineralocorticoid hormone-. Competitively inhibits mineralocorticoid receptors by blocking the mineralocorticoid receptor, the spironolactone inhibits the effects of mineralocorticoids in the body and increases levels of aldosterone.	Spironolactone is well absorbed after oral administration, with peak plasma concentration reached approximately 2 hours after ingestion. It undergoes extensive hepatic metabolism and has an average elimination half-life of about 1.4 hours. Spironolactone and its metabolites are primarily excreted in the urine.	Used for the treatment of hypertension, hyperaldosteronism, edema due to various conditions, hirsutism, and hypokalemia.

*Note*. Pharmacodynamics and Pharmacokinetics of each drug used, and the use related to medical conditions. From "*Drugbank online database*", by Wishart et al. (2006). https://go.drugbank.com/

