# Supplementary material for: Do stress hormones influence choice? A systematic review of pharmacological interventions on the HPA axis and/or SAM system
Source: Soc Cogn Affect Neurosci. 2024 Oct 4;19(1):nsae069. doi: 10.1093/scan/nsae069 (PMC11498176; doi:10.1093/scan/nsae069)
Supplement: nsae069_Supp [file nsae069_supp.zip › scan-22-178-File006.docx]

The effect of stress neuromodulators administration in decision-making: a systematic review

Supplementary figures


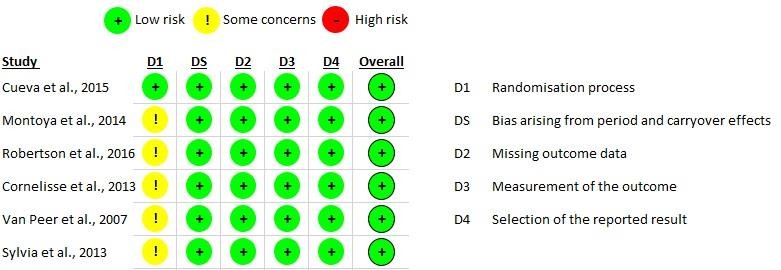
**Supplementary Figure 1.** *Risk of bias analysis* **–** ROB 2.

# A


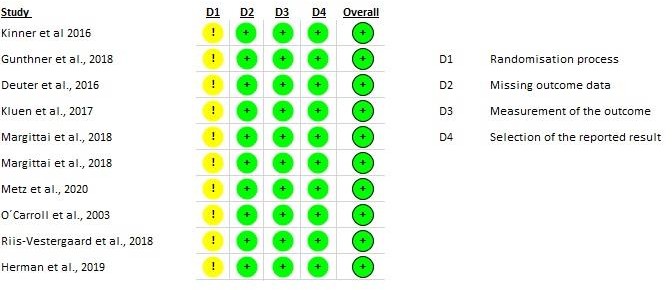
**B**

**Note**: RoB 2 for parallel trials, RoB 2 for crossover trials and ROBINS I for non-randomized trials. For RoB 2, most of the studies present a low risk of bias in the revised domains (**A** and **B**).

**Supplementary Figure 2.** *Risk of bias analysis* **–** ROBINS I.


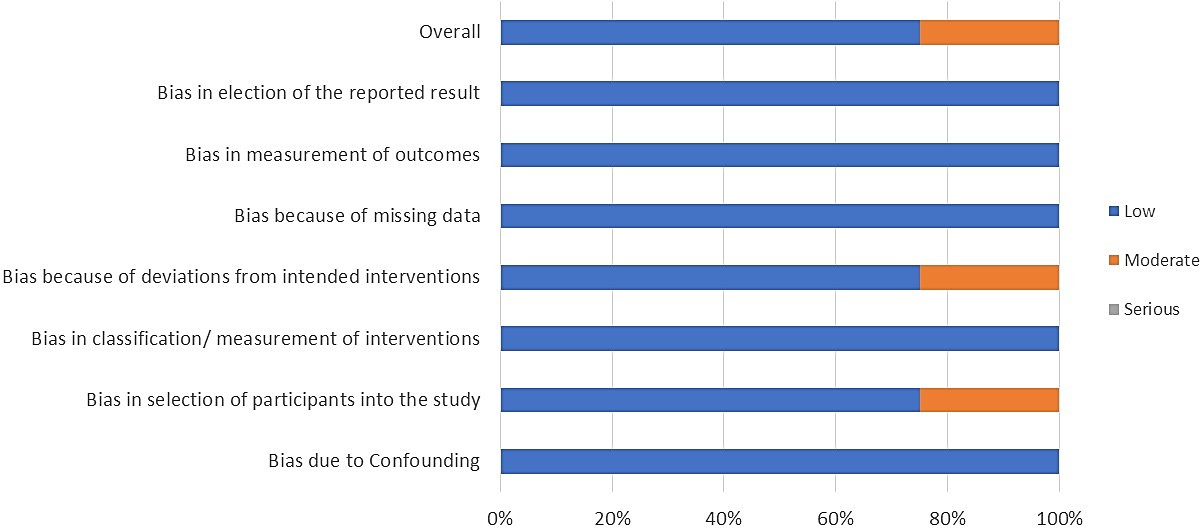


**Note**: in ROBINS I, most of the studies presented low risk of bias except for one study that presented moderate risk of bias in the domain selection of the participants into the study.

| **Section and Topic** | **Item #** | **Checklist item** | **Location where item**  **is reported** |
| --- | --- | --- | --- |
| **TITLE** | | |  |
| Title | 1 | Identify the report as a systematic review. | 1 |
| **ABSTRACT** | | |  |
| Abstract | 2 | See the PRISMA 2020 for Abstracts checklist. | 2 |
| **INTRODUCTION** | | |  |
| Rationale | 3 | Describe the rationale for the review in the context of existing knowledge. | 2-3 |
| Objectives | 4 | Provide an explicit statement of the objective(s) or question(s) the review addresses. | 3 |
| **METHODS** | | |  |
| Eligibility criteria | 5 | Specify the inclusion and exclusion criteria for the review and how studies were grouped for the syntheses. | 4 |
| Information sources | 6 | Specify all databases, registers, websites, organisations, reference lists and other sources searched or consulted to identify studies. Specify the date when each source was last searched or consulted. | 4 |
| Search strategy | 7 | Present the full search strategies for all databases, registers and websites, including any filters and limits used. | 4 |
| Selection process | 8 | Specify the methods used to decide whether a study met the inclusion criteria of the review, including how many reviewers screened each record and each report retrieved, whether they worked independently, and if applicable, details of automation tools used in the process. | 4 |
| Data collection process | 9 | Specify the methods used to collect data from reports, including how many reviewers collected data from each report, whether they worked independently, any processes for obtaining or confirming data from study investigators, and if applicable, details of automation tools used in the process. | 4 |
| Data items | 10a | List and define all outcomes for which data were sought. Specify whether all results that were compatible with each outcome domain in each study were sought (e.g. for all measures, time points, analyses), and if not, the methods used to decide which results to collect. | 4 |
|  | 10b | List and define all other variables for which data were sought (e.g. participant and intervention characteristics, funding sources). Describe any assumptions made about any missing or unclear information. | 4 |
| Study risk of bias assessment | 11 | Specify the methods used to assess risk of bias in the included studies, including details of the tool(s) used, how many reviewers assessed each study and whether they worked independently, and if applicable, details of automation tools used in the process. | 5 |
| Effect measures | 12 | Specify for each outcome the effect measure(s) (e.g. risk ratio, mean difference) used in the synthesis or presentation of results. | 7 |
| Synthesis methods | 13a | Describe the processes used to decide which studies were eligible for each synthesis (e.g. tabulating the study intervention characteristics and comparing against the planned groups for each synthesis (item #5)). | 4 |
|  | 13b | Describe any methods required to prepare the data for presentation or synthesis, such as handling of missing summary statistics, or data conversions. | 6 |
|  | 13c | Describe any methods used to tabulate or visually display results of individual studies and syntheses. | 6 |
|  | 13d | Describe any methods used to synthesize results and provide a rationale for the choice(s). If meta-analysis was performed, describe the model(s), method(s) to identify the presence and extent of statistical heterogeneity, and software package(s) used. | 14 |
|  | 13e | Describe any methods used to explore possible causes of heterogeneity among study results (e.g. subgroup analysis, meta-regression). | - |
|  | 13f | Describe any sensitivity analyses conducted to assess robustness of the synthesized results. | - |
| Reporting bias assessment | 14 | Describe any methods used to assess risk of bias due to missing results in a synthesis (arising from reporting biases). | 5 |
| Certainty assessment | 15 | Describe any methods used to assess certainty (or confidence) in the body of evidence for an outcome. | - |

| **Section and Topic** | **Item #** | **Checklist item** | **Location where item**  **is reported** |
| --- | --- | --- | --- |
| **RESULTS** | | |  |
| Study selection | 16a | Describe the results of the search and selection process, from the number of records identified in the search to the number of studies included in the review, ideally using a flow diagram. | 5 |
|  | 16b | Cite studies that might appear to meet the inclusion criteria, but which were excluded, and explain why they were excluded. | 5 |
| Study characteristics | 17 | Cite each included study and present its characteristics. | 6 |
| Risk of bias in studies | 18 | Present assessments of risk of bias for each included study. | 11 |
| Results of individual studies | 19 | For all outcomes, present, for each study: (a) summary statistics for each group (where appropriate) and (b) an effect estimate and its precision (e.g. confidence/credible interval), ideally using structured tables or plots. | 7-10 (Table  1) |
| Results of syntheses | 20a | For each synthesis, briefly summarise the characteristics and risk of bias among contributing studies. |  |
|  | 20b | Present results of all statistical syntheses conducted. If meta-analysis was done, present for each the summary estimate and its precision (e.g. confidence/credible interval) and measures of statistical heterogeneity. If comparing groups, describe the direction of the effect. | 12 |
|  | 20c | Present results of all investigations of possible causes of heterogeneity among study results. | 12-15 |
|  | 20d | Present results of all sensitivity analyses conducted to assess the robustness of the synthesized results. | - |
| Reporting biases | 21 | Present assessments of risk of bias due to missing results (arising from reporting biases) for each synthesis assessed. | - |
| Certainty of evidence | 22 | Present assessments of certainty (or confidence) in the body of evidence for each outcome assessed. | - |
| **DISCUSSION** | | |  |
| Discussion | 23a | Provide a general interpretation of the results in the context of other evidence. | 15 |
|  | 23b | Discuss any limitations of the evidence included in the review. | 21 |
|  | 23c | Discuss any limitations of the review processes used. | 21 |
|  | 23d | Discuss implications of the results for practice, policy, and future research. | 20-21 |
| **OTHER INFORMATION** | | |  |
| Registration and protocol | 24a | Provide registration information for the review, including register name and registration number, or state that the review was not registered. | 3 |
|  | 24b | Indicate where the review protocol can be accessed, or state that a protocol was not prepared. | 3 |
|  | 24c | Describe and explain any amendments to information provided at registration or in the protocol. | 3 |
| Support | 25 | Describe sources of financial or non-financial support for the review, and the role of the funders or sponsors in the review. | 21 |
| Competing interests | 26 | Declare any competing interests of review authors. | 22 |
| Availability of data, code and other materials | 27 | Report which of the following are publicly available and where they can be found: template data collection forms; data extracted from included studies; data used for all analyses; analytic code; any other materials used in the review. | - |

*From:* Page MJ, McKenzie JE, Bossuyt PM, Boutron I, Hoffmann TC, Mulrow CD, et al. The PRISMA 2020 statement: an updated guideline for reporting systematic reviews. BMJ 2021;372:n71. doi: 10.1136/bmj.n71

# Search Criteria

| Table 1. Full Search criteria for databases | | |
| --- | --- | --- |
| **Keywords** | **Numeration** | **Search criteria** |
| Hydrocortisone | 1. | MeSH Hydrocortisone descriptor (this term only) (Title/Abstract) |
|  | 2. | MeSH Fludrocortisone descriptor (this term only) (Title/Abstract) |
|  | 3. | #1 OR #2 |
| Norepinephrine | 4. | MeSH Norepinephrine descriptor (this term only) (Title/Abstract) |
|  | 5. | MeSH Noradrenaline descriptor (this term only) (Title/Abstract) |
|  | 6. | #4 OR #5 |
|  | 7. | #3 OR #6 |
| Cortisol | 8. | MeSH Cortisol descriptor (this term only) (Title/Abstract) |
|  | 9. | #3 OR #7 OR #8 |
| Pharmacological-induced Stress | 10. | Pharmacological-induced Stress (Title/Abstract) |
|  | 11. | Stress, Pharmacological-induced |
|  | 12. | Pharmacological-induced Stress (Title/Abstract) |
|  | 13. | MeSH Glucocorticoids (this term only) (Title/Abstract) |
|  | 14. | #10 OR #11 OR #12 OR #13 |
|  | 15. | #3 OR #7 OR #9 OR #14 |
| Acute Stress | 16. | Acute Stress (Title/Abstract) |
|  | 17. | Stress, Acute |
|  | 18. | #15 OR #16 |
|  | 19. | #3 OR #7 OR #9 OR #14 OR #18 |
| Decision Making | 20. | MeSH Decision-Making descriptor (this term only) (Title/Abstract) |
|  | 21. | MeSH Temporal Discounting descriptor (this term only) (Title/Abstract) |
|  | 22. | MeSH Decision-making under risk descriptor (this term only) (Title/Abstract) |
|  | 23. | MeSH Moral decision-making descriptor (this term only) (Title/Abstract) |
|  | 24. | MeSH Social decision-making descriptor (this term only) (Title/Abstract) |
|  | 25. | #19 OR #20 OR #21 OR #22 OR #23 |
|  | 26. | #3 OR #7 OR #9 OR #14 OR #18 AND #25 |

(((((((((((((hydrocortisone[MeSH Terms]) OR (Hydrocortisone[Title/Abstract])) OR (Fludrocortisone[Title/Abstract])) OR **Pubmed**

(Fludrocortisone[MeSH Terms])) OR (Norepinephrine[MeSH Terms])) OR (Norepinephrine[Title/Abstract])) OR (Cortisol[MeSH Terms])) OR (Cortisol[Title/Abstract])) OR (pharmacologically induced stress[Title/Abstract])) OR (Acute Stress[Title/Abstract]))) ) OR (Glucocorticoids[MeSH Terms])) OR (Glucocorticoids[Title/Abstract]) AND (((((((((((Decision-Making[MeSH Terms]) OR (Decision-Making[Title/Abstract])) OR (Temporal Discounting[MeSH Terms])) OR (Temporal Discounting[Title/Abstract])) OR (Decision-making under risk[MeSH Terms])) OR (Decision-making under risk[Title/Abstract])) OR (Moral Decision-making[MeSH Terms])) OR (Moral Decision-making[Title/Abstract])) OR (Social Decision-making[MeSH Terms])) OR (Social decision-making[Title/Abstract])))

# APA

MeSH: Hydrocortisone descriptor OR Any Field: Hydrocortisone descriptor OR MeSH: Fludrocortisone OR Any Field: Fludrocortisone OR MeSH: Norepinephrine OR Any Field: Norepinephrine OR MeSH: Cortisol OR Any Field: Cortisol OR Any Field: Pharmacological-induced Stress OR Any Field: Stress, Pharmacological-induced OR MeSH: Glucocorticoids OR Any Field: Glucocorticoids OR Any Field: Acute Stress OR Any Field: Stress, Acute AND MeSH: Decision-Making OR Any Field: Decision-Making OR MeSH: Temporal Discounting OR Any Field: Temporal Discounting OR MeSH: Moral decision-making OR Any Field: Moral decision-making OR MeSH: Social decision-making OR Any Field: Social decision-making

# Web of Science

ALL=("Decision-Making") OR ALL=("Temporal Discounting") OR ALL=("Risk Decision-making") OR ALL=("Moral decision-making") OR ALL=("Social decision-making") AND ALL=(Hydrocortisone) OR ALL=(Fludrocortisone) OR ALL=(Norepinephrine) OR ALL=(Noradrenaline) OR ALL=(Cortisol) OR ALL=("Pharmacological-induced Stress") OR ALL=(Glucocorticoids) OR ALL=("Acute Stress")

# Scopus

( ( TITLE-ABS-KEY ( hydrocortisone ) OR TITLE-ABS-KEY ( fludrocortisone ) OR TITLE-ABS-KEY ( norepinephrine ) OR TITLE-ABS-KEY ( noradrenaline ) OR TITLE-ABS-KEY ( cortisol ) OR TITLE-ABS-KEY ( "Pharmacological-induced Stress" ) OR TITLE-ABS-KEY ( glucocorticoids

) OR TITLE-ABS-KEY ( "Acute Stress" ) ) ) AND ( ( TITLE-ABS-KEY ( "Decision-Making" ) OR TITLE-ABS-KEY ( "Temporal Discounting" ) OR TITLE-ABS-KEY ( "Decision-making" ) OR TITLE-ABS-KEY ( "Moral decision" ) OR TITLE-ABS-KEY ( "Social decision" ) ) ) AND ( LIMIT-TO ( DOCTYPE , "ar" ) )

# Google Academic

"decision making" AND Cortisol -review -animal -book -letter -editor -chapter -children -patient -protocol -disease

# Cochrane Library

(Hydrocortisone):ti,ab,kw OR (Glucocorticoids):ti,ab,kw OR (Norepinephrine):ti,ab,kw OR (Acute Stress):ti,ab,kw OR (Cortisol):ti,ab,kw AND (Hydrocortisone):ti,ab,kw OR (Glucocorticoids):ti,ab,kw OR (Norepinephrine):ti,ab,kw OR (Acute Stress):ti,ab,kw OR (Cortisol):ti,ab,kw

# ScienceDirect

"Cortisol" AND "Decision-making"

# Eligibility criteria

What is the effect of cortisol administration in decision-making? P: Healthy Adults

I: Cortisol administration

C:Control (Placebo. A group who were not exposed to the pharmacological intervention) O: Decision-making

1. The review is looking for the influence of stress neuromodulator administration in human decision-making. Some studies mention that stress affects the psychobiology behind cognition, behavior, and emotion. Moreover, there are reports of neurobiological changes associated with stress, specifically in neurotransmitters and hormones.
2. Inclusions: Healthy adults

Exclusion: People under 18 years old, and people with a mental or organic condition.

1. Many decisions are made under physical or emotional stress. Stress can be defined as an individual's response to a stressor, that is, a condition that challenges or even exceeds the resources of the individual. Cortisol administration elicit the stress response.
2. The type of studies included will be research articles that include the PICO question. There will be excluded book chapters, letters to the editor, books, animal studies, study cases, pilot studies and protocols, systematic reviews, meta-analysis.
3. The primary outcomes represent any measure with information about the relationship between the cortisol administration associated with decision- making.
